# Urokinase vs Tissue-Type Plasminogen Activator for Thrombolytic Evacuation of Spontaneous Intracerebral Hemorrhage in Basal Ganglia

**DOI:** 10.3389/fneur.2017.00371

**Published:** 2017-08-03

**Authors:** Yuqian Li, Ruixin Yang, Zhihong Li, Bo Tian, Xingye Zhang, Jiancai Wang, Longlong Zheng, Boliang Wang, Lihong Li

**Affiliations:** ^1^Department of Neurosurgery, Tangdu Hospital, The Fourth Military Medical University, Xi’an, Shaanxi, China; ^2^Department of Emergency, Tangdu Hospital, The Fourth Military Medical University, Xi’an, Shaanxi, China

**Keywords:** intracerebral hemorrhage, thrombolytic evacuation, urokinase, tissue-type plasminogen activator, basal ganglia

## Abstract

Spontaneous intracerebral hemorrhage (ICH) is a devastating form of stroke, which leads to a high rate of mortality and poor neurological outcomes worldwide. Thrombolytic evacuation with urokinase-type plasminogen activator (uPA) or tissue-type plasminogen activator (tPA) has been showed to be a hopeful treatment for ICH. However, to the best of our knowledge, no clinical trials were reported to compare the efficacy and safety of these two fibrinolytics administrated following minimally invasive stereotactic puncture (MISP) in patients with spontaneous basal ganglia ICH. Therefore, the authors intended here to evaluate the differential impact of uPA and tPA in a retrospective study. In the present study, a total of 86 patients with spontaneous ICH in basal ganglia using MISP received either uPA (uPA group, *n* = 45) or tPA (tPA group, *n* = 41), respectively. The clinical baseline characteristics prior to the operation were collected. In addition, therapeutic responses were assessed by the short-term outcomes within 30 days postoperation, as well as long-term outcomes at 1 year postoperation. Our findings showed that, in comparison with tPA, uPA was able to better promote hematoma evacuation and ameliorate perihematomal edema, but the differences were not statistically significant. Moreover, the long-term functional outcomes of both groups were similar, with no statistical difference. In conclusion, these results provide evidence supporting that uPA and tPA are similar in the efficacy and safety for thrombolytic evacuation in combination with MISP in patients with spontaneous basal ganglia ICH.

## Introduction

Intracerebral hemorrhage (ICH) is one of the most devastating subtypes of cerebrovascular diseases with extremely high mortality, severe disability in the surviving patients and little effective treatment (1–3). According to epidemiological reports, ICH remains the second most common form of stroke, accounting for 10–15% of all strokes, with an incidence of 10–30 per 100,000 people each year worldwide (4, 5). Besides, approximately 50–70% of all spontaneous ICH is located in the area of the basal ganglia. Hitherto, the 30-day mortality rate of ICH is 35–52% with half of such deaths occurring in the first 2 days, and only 20% of those surviving victims are independent within 6 months (6, 7). The high mortality and morbidity are closely associated with age, unstable blood pressure, hematoma volume (HV), perihematomal edema (PHE), rebleeding, intraventricular extension, hematoma location, presenting level of consciousness, as well as secondary brain injury following the initial hemorrhage in the subsequent days (8, 9).

Over the last decades, controversy still continues about the management of spontaneous ICH. Compared with conservative medical treatment, attempts at hematoma removal *via* conventional aggressive surgical treatment have failed to show more benefits for most ICH patients, reflecting an uncertainty about the effect of surgery (2, 10). However, a powerful evidence provided by the results of Surgery for Primary Supratentorial Intracerebral Hemorrhage demonstrated that surgery plus medical management significantly reduced the mortality and morbidity at final follow-up when compared to routine medical management alone in patients with supratentorial ICH (11). Accumulating evidence has frequently supported the role of two structurally different fibrinolytic agents, urokinase-type plasminogen activator (uPA), and tissue-type plasminogen activator (tPA), for clot lysis in the treatment of ICH, indicating that both fibrinolytics could accelerate the intracranial hematoma fibrinolysis of patients suffering from ICH (12). More importantly, it is reported by several studies that the administration of tPA following minimally invasive stereotactic puncture (MISP) might speed up clot lysis, decrease PHE, and improve long-term outcomes with an acceptable safety profile (13–16). To the best of our knowledge, however, no clinical trials have been carried out to compare the differential effect of these two fibrinolytic agents administrated following MISP in patients with spontaneous basal ganglia ICH. We hypothesized that there were no differences in efficacy and safety profile between uPA and tPA in such condition. Thus, we decided to test this hypothesis in a retrospective clinical trial.

## Materials and Methods

### Subjects

A single-center retrospective clinical trial was carried out in the department of neurosurgery, the second affiliated hospital of Fourth Military Medical University. According to the inclusion and exclusion criteria, a total of 86 eligible in-hospital patients (47 males, 39 females) who were diagnosed with spontaneous ICH in basal ganglia from January 2014 to June 2015 were enrolled in the clinical trial. 45 individuals who received MISP + uPA were assigned to the uPA group, and the remaining 41 individuals with MISP + tPA were assigned to the tPA group. Patient characteristics at admission, including age, gender, systolic blood pressure (SBP), HV, direction of hematoma, Acute Physiology and Chronic Health Evaluation (APACHE) II, Glasgow coma scale (GCS) score, ICH score, the number of patients with intraventricular hemorrhage (IVH), history of diabetes, and hypertension were collected from each patient. All procedures performed in the study were approved by the Institutional Investigational Review board at the Fourth Military Medical University.

### Inclusion and Exclusion Criteria

Inclusion criteria for patients in the present study were as follows: (1) spontaneous ICH in the basal ganglia (right or left); (2) HV ≥ 30 mL; (3) GCS score ≥ 4; (4) age range: 18–80 years; (5) hematoma evacuation using MISP within 24 h of ictus.

Exclusion criteria for patients in the present study were as follows: (1) ICH caused by intracranial aneurysms, tumors, arteriovenous malformation, trauma or infraction; (2) signs of cerebral hernia or death; (3) patients with coagulopathy; (4) patients with intracranial, pulmonary, or general infection; (5) a prior stroke history with neurological deficits; (6) any previous history of severe heart failure, renal failure, hepatic or pulmonary dysfunctions; and (7) female patients in pregnancy or lactation.

### Analysis of the Volume of Hematoma and PHE

All the volume measurements of intraparenchymal clot and cerebral edema were performed by WAM and JRC using an open source DICOM software program for Mac (OsiriX v. 4.1, Pixmeo, Geneva, Switzerland). The suspicious positive area of ICH and PHE were manually drawn for further computerized analysis. To identify regions of PHE, we used a semi-automated threshold-based method using 5–33 HU, which was reported by Volbers. 5 HU was fixed as the lower value, while the upper limit of 33 HU was adjusted to obtain the best delineation of edema area and avoid artifact introduced by leukoaraiosis. Once HU limits were set, Osirix could calculate the volume of the edema regions through computing ROI and slice thickness. The similar threshold-based method was used to calculate volumes of blood with a clear boundary on CT.

### General Care

All enrolled patients received the standard medical management according to the guidelines for the treatment of spontaneous ICH in adults from the American Heart Association/American Stroke Association Stroke Council, high blood pressure research council, and the quality of care and outcomes in research interdisciplinary working group (17). SBP was decreased and maintained at ≤140 mmHg, with cerebral perfusion pressure above 70 mmHg. Besides, central venous pressure was evaluated and maintained between 4 and 12 cmH_2_O to avoid hypervolemia or hypovolemia. The core temperature, electrolyte, blood glucose values were kept in the normal range. Arterial blood gas analysis was monitored at least once a day. In particular, critically ill patients need immediate respiratory support with mechanical ventilation when GCS scores < 9 or a rapid deterioration in neurological status occurs. Additionally, treatments also included nutritional support, sedation and analgesia therapy, control of cerebral edema, and the prevention of complications.

### MISP and Thrombolysis Therapy

The procedures were similar with the method described by Mould et al. (18). All operations were performed under general anesthesia. Using the stereotactical frame (Gaoxun Company, Guangzhou, Guangdong, China), a 10-French cannula was placed by stereotactic means into the hemorrhage clot according to preoperative CT, with the tip at two-thirds the length of the long axis. Special attention was paid to avoid injuring important cortical function areas and main blood vessels. After confirming the positioning of a cannula with postoperative CT within 24 h postoperatively, intraclot administration of tPA (1 mg, Actilyse, Boehringer Ingelheim International) or urokinase (1.0 × 10^4^ IU, Ndpharm, Nanjing, Jiangsu, China) was performed every 8 h, up to nine doses, or until the residue hematoma was less than 10 mL. Of note, treatments should be ended when any significant recurrence of ICH events or any new hemorrhage occurs. The dose regimen of tPA is based on a preliminary report of the clot lysing accelerated resolution of IVH (CLEAR-IVH) clinical trial (19), suggesting that the right dose for tPA was supposed to be 1 mg three times a day. For uPA, no acccurate dose has been recommended as yet. Thus, the dose regimen in the trial was in accordance with the report from Gaberel et al. who reviewed the relevant literature and considered that 1.0 × 10^4^ IU of uPA is equivalent to 1 mg of tPA (12). After each assigned dose, the system was closed for 1 h to allow clot and drug interaction. After that, the system was opened again for gravitational drainage. CT scans were obtained every 24 h to evaluate drainage, or as clinically indicated.

### Follow-up, Short-term, and Long-term Outcome Assessment

All enrolled patients followed identical assessment criteria and methods. The baseline characteristics at admission, including age, gender, history of diabetes and hypertension, SBP, APACHE II, GCS score, and HV were assessed. The short-term outcomes within 30 days after ictus was assessed according to GCS score, APACHE II, residue hematoma, evacuation rate (ER), PHE, recurrence of ICH events, frequency of drug administration, catheter-retained time, duration of hospitalization, total expenditure, and the incidence of complications. Functional independence was assessed at 1 year after stroke using the mortality, Glasgow Outcome Scale (GOS), Barthel index (BI), and modified Rankin Scale (mRS). The data of all evaluation parameters for both groups were analyzed and compared. The follow-up information of all the patients was completed *via* telephone or an interview with the patient or their family.

### Statistics Analysis

The data collected in the present study were analyzed with the SPSS 20.0 software (SPSS Inc., Chicago, IL, USA). Categorical variables were analyzed by the Pearson’s Chi-square test. Continuous variables were verified for normality using the Kolmogorov–Smirnov test. Normally distributed data were represented as mean ± SD, and the comparison of variables between both groups was performed by independent sample *t* test. By contrast, non-parametric data were analyzed using the Kruskal–Wallis test. For all analyses, *p*-value less than 0.05 was considered statistically significant.

## Results

### General Results

A total of 86 neurosurgical patients (47 males, 39 females) were recruited according to the inclusion and exclusion criteria in this study. Of these, 45 subjects received uPA following MISP, while the other 41 subjects received tPA. Baseline characteristics of patients in the uPA and tPA groups were similar, with no significant differences observed. Overall, female patients accounted for 46.7 and 48.8% in the uPA and tPA group, respectively (*p* = 0.845). The mean age was 59.8 ± 9.0 years for uPA-treated patients, while 56.7 ± 11.2 years for tPA-treated patients (*p* = 0.336). Moreover, both the mean HV [54.2 ± 13.2 mL (uPA) vs 56.7 ± 11.2 mL (tPA), *p* = 0.556] and SBP [159.8 ± 24.8 mmHg (uPA) vs 162.1 ± 20.7 mmHg (tPA), *p* = 0.766] were similar between the two groups, with no statistical difference. Further, no statistically significant difference was observed in APACHE II [35.5 ± 9.5 (uPA) vs 34.1 ± 9.7 (tPA), *p* = 0.645], GCS score [8.9 ± 2.6 (uPA) vs 9.4 ± 2.9 (tPA), *p* = 0.595], or ICH score [2.6 ± 0.7 (uPA) vs 2.4 ± 0.6 (tPA), *p* = 0.266]. Finally, there was no statistical difference between both groups in the direction of hematoma, the number of patients with IVH, and history of diabetes and hypertension (Table 1).

**Table 1:** Clinical baseline characteristics of patients.

Baseline characteristics	Urokinase-type plasminogen activator (45)	Tissue-type plasminogen activator (41)	*p*-Value
Female, *n* (%)	21 (46.7)	20 (48.8)	0.845
Age (years)	59.8 ± 9.0	56.7 ± 11.2	0.336
SBP (mmHg)	159.8 ± 24.8	162.1 ± 20.7	0.766
HV (mL)	54.2 ± 13.2	56.7 ± 11.2	0.556
Direction (left/right)	24/21	19/22	0.517
GCS (score)	8.9 ± 2.6	9.4 ± 2.9	0.595
APACHE II (score)	35.5 ± 9.5	34.1 ± 9.7	0.645
Intracerebral hemorrhage score (score)	2.6 ± 0.7	2.4 ± 0.6	0.266
IVH, *n* (%)	8 (17.8)	9 (21.9)	0.627
Diabetes, *n* (%)	8 (17.8)	6 (14.6)	0.693
Hypertension, *n* (%)	31 (68.9)	30 (73.2)	0.662

*SBP, systolic blood pressure; HV, hematoma volume; GCS, Glasgow Coma Scale; APACHE II, Acute Physiology and Chronic Health Evaluation II; IVH, intraventricular hemorrhage*.

### Short-term Outcomes

**Figure 1:**
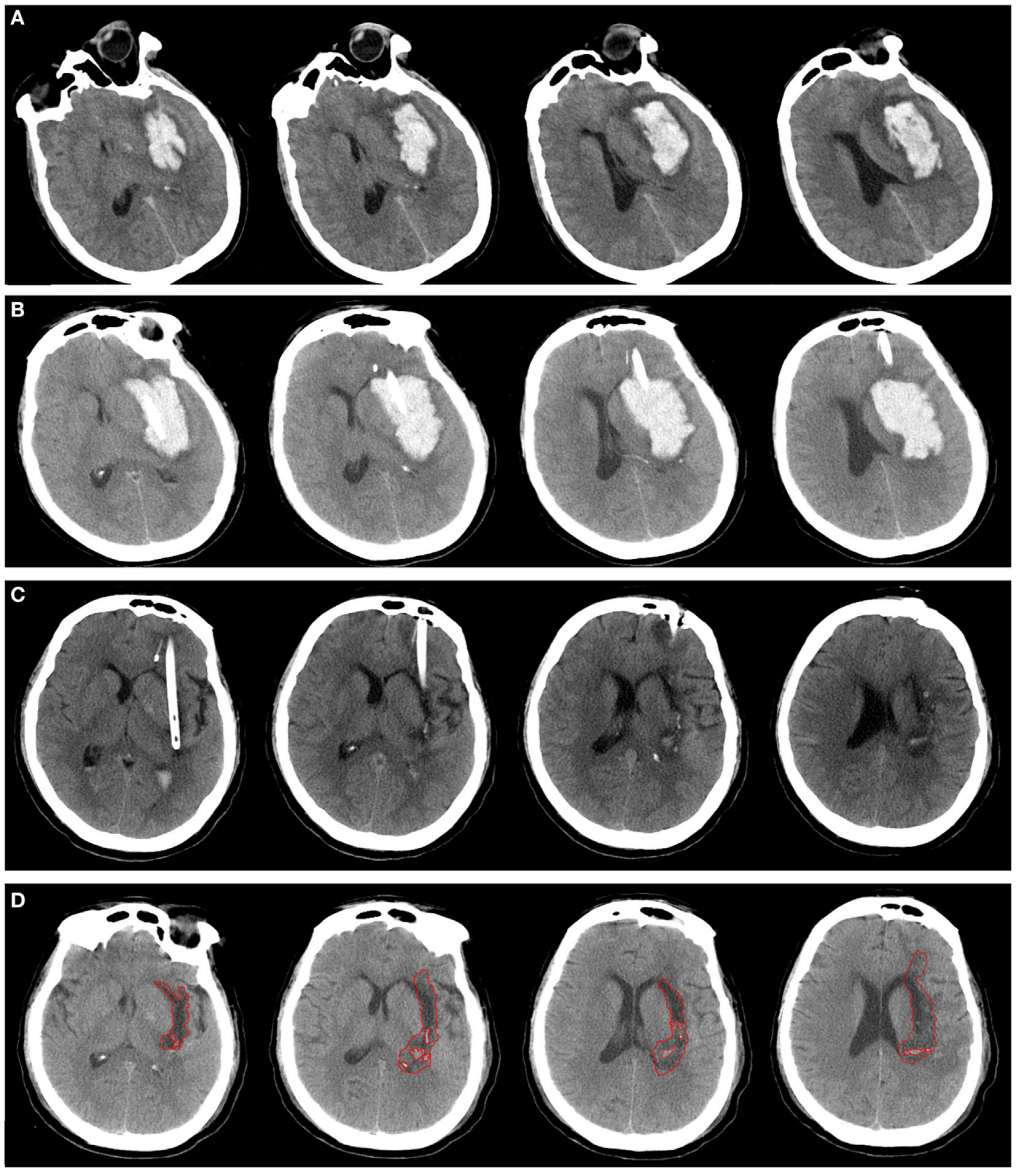
A patient with spontaneous intracerebral hemorrhage in basal ganglia received urokinase-type plasminogen activator (uPA) at 24 h postoperatively. Computed tomography scan before operation **(A)**, at postoperative day 1 **(B)**, after application of uPA **(C)**, and postictus day 7 **(D)**. The perihematomal edema was semiautomatically threshold-based segmentated and outlined with red line **(D)**.

**Figure 2:**
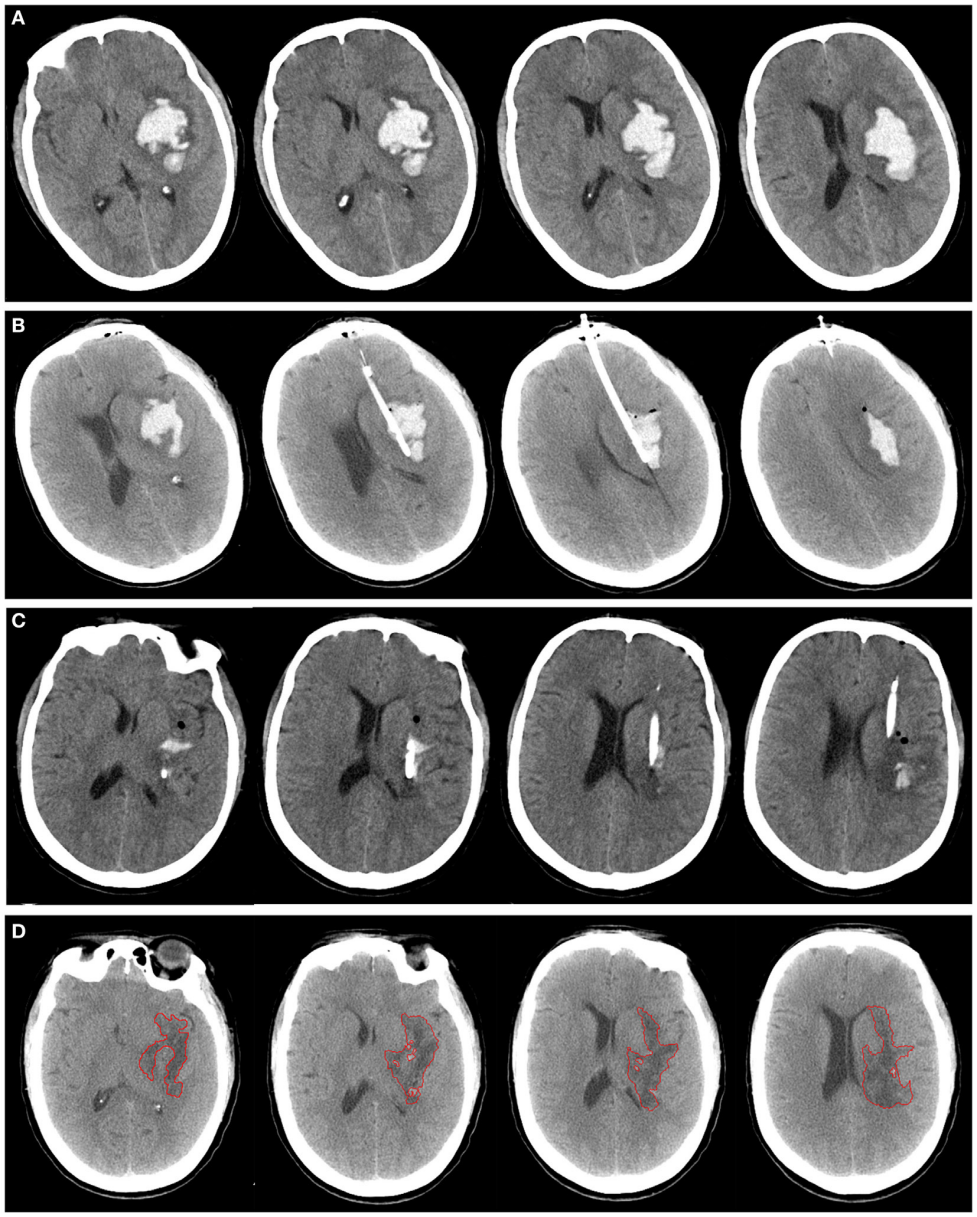
A patient with spontaneous intracerebral hemorrhage in basal ganglia received tissue-type plasminogen activator (tPA) at 24 h postoperatively. Computed tomography scan before operation **(A)**, at postoperative day 1 **(B)**, after application of tPA **(C)**, and postictus day 7 **(D)**. The perihematomal edema was semiautomatically threshold-based segmentated and outlined with red line **(D)**.

As shown in Table 2, short-term outcomes within 30 days after operation was assessed according to HV, PHE, recurrence of rebleeding, and the incidence of complications. The residual HV on day 1 postoperation in both groups was not significantly different [31.3 ± 8.9 mL (uPA) vs 33.8 ± 9.1 mL (tPA), *p* = 0.396], as well as the ER [41.9 ± 10.3% (uPA) vs 40.7 ± 8.8% (tPA), *p* = 0.711]. After application of the two drugs, the residual HV in uPA-treated patients (9.7 ± 4.9 mL) was slightly less than that in tPA-treated patients (11.6 ± 6.5 mL), but the differences between the two groups were not statistically significant (*p* = 0.296). And the ER in the uPA group was higher than that in the tPA group [82.6 ± 7.9% (uPA) vs 79.9 ± 9.5% (tPA), *p* = 0.332], showing a comparable fibrinolytic effect in ICH fibrinolysis. Then, the volume of PHE was examined on Day 7 after surgery, with 66.1 ± 22.4 mL for uPA-treated patients, corresponding to 68.9 ± 19.1 mL for tPA-treated individuals. Although the volume was larger in the tPA group, the difference between both groups was not statistically significant (*p* = 0.687). Further, the times of usage and catheter-retained time were investigated, and we found that there was no significant difference between both groups in either the times of usage [5.6 ± 2.1 (uPA) vs 5.4 ± 2.2 (tPA), *p* = 0.829] or catheter-retained time [42.9 ± 13.9 h (uPA) vs 40.4 ± 12.4 h (tPA), *p* = 0.552]. Moreover, 2 subjects in the uPA group suffered from recurrence of ICH, accounting for 4.4% of all uPA-treated patients, which showed no significant difference with the value obtained for tPA-treated patients (4.9%, 2/41) (*p* = 0.924). In addition, no significant difference was observed between both groups in the incidence of postoperative and drug-related complications, including gastrointestinal bleeding, renal failure, intracranial infection, pulmonary infection, and epilepsy. The length of hospitalization was indistinguishable between the two groups, while the total expenditure was much higher for tPA-treated patients than that for uPA-treated patients (*p* = 0.029) (Figures 1 and 2).

**Table 2:** Short-term outcomes within 30 days postoperation.

Clinical outcomes	Urokinase-type plasminogen activator (45)	Tissue-type plasminogen activator (41)	*p*-Value
Residue HV on day 1 (mL)	31.3 ± 8.9	33.8 ± 9.1	0.396
ER on day 1 (%)	41.9 ± 10.3	40.7 ± 8.8	0.711
Residue HV after application of agents (mL)	9.7 ± 4.9	11.6 ± 6.5	0.296
ER after application of agents (%)	82.6 ± 7.9	79.9 ± 9.5	0.332
PHE on day 7 (mL)	66.1 ± 22.4	68.9 ± 19.1	0.687
Times of usage (times)	5.4 ± 2.2	5.6 ± 2.1	0.829
Catheter-retained time (h)	40.4 ± 12.4	42.9 ± 13.9	0.552
Recurrence of ICH, *n* (%)	2 (4.4)	2 (4.9)	0.924
Gastrointestinal bleeding, *n* (%)	5 (11.1)	4 (9.8)	0.838
Renal failure, *n* (%)	1 (2.2)	1 (2.4)	0.947
Intracranial infection, *n* (%)	2 (4.4)	1 (2.4)	0.613
Pulmonary infection, *n* (%)	4 (8.7)	3 (7.3)	0.790
Epilepsy, *n* (%)	7 (15.6)	5 (12.2)	0.653
Hospitalization length (day)	9.2 ± 2.9	9.4 ± 2.3	0.862
Total expenditure (¥, thousand Yuan)	42.6 ± 5.4	47.0 ± 6.2	0.029

*HV, hematoma volume; ER, evacuation rate; PHE, perihematomal edema; ICH, intracerebral hemorrhage*.

### Long-term Outcomes

After 1 year of follow-up, long-term outcomes, including mortality, GOS, BI, and mRS, were further analyzed and showed in Table 3. The total mortality in the present study was 12.8% (11/86), with 13.3% (6/45) and 12.2% (5/41) in the uPA and tPA group, respectively, and the difference between both group was not significant (*p* = 0.875). GOS, BI, and mRS were collected and compared between uPA and tPA group. The data showed that all these three parameters between both groups were not statistically significant (GOS, *p* = 0.178; BI, *p* = 0.434; and mRS, *p* = 0.491, respectively).

**Table 3:** Long-term outcomes 1 year after ictus.

Clinical outcomes	Urokinase-type plasminogen activator (45)	Tissue-type plasminogen activator (41)	*p*-Value
Case fatality, *n* (%)	6 (13.3)	5 (12.2)	0.875
GOS	4.1 ± 0.7	3.7 ± 0.9	0.178
BI	80.6 ± 17.8	76.3 ± 14.1	0.434
mRS	2.1 ± 1.0	2.3 ± 1.2	0.491

*GOS, Glasgow Outcome Scale; BI, Barthel index; mRS, modified Rankin Scale*.

## Discussion

The mass effect caused by intracranial hematoma and secondary PHE could contribute to brain damage, such as intracranial hypertension or cerebral hernia, which is significantly associated with mortality, morbidity, and poor functional outcomes after ICH (20, 21). Fibrinolytic therapy has been confirmed to play an important role in accelerating the lysis and elimination of hematoma, alleviating brain edema, and improving the prognosis in patients with ICH (22). In the present study, we investigated and compared, for the first time, the efficacy and safety of uPA and tPA administrated following MISP in patients with spontaneous basal ganglia ICH. Consistent with previous study performed in a model of ICH in rats, our findings demonstrated that administration of uPA exhibited a similar therapeutic effect on both short-term and long-term clinical outcomes with tPA.

In clinical practice, tPA and uPA have been widely used for thrombolysis in acute pulmonary embolism, acute ischemic stroke, and acute myocardial infarction (23, 24). Over the past few decades, with the progress of modern stereotactic and image guided neurosurgical techniques, some small clinical trials of minimal invasive puncture and drainage in combination with thrombolytics have obtained satisfactory therapeutic benefits with an acceptable safety profile (25–28). However, the application to ICH is still limited, due to the unstandardized dosage and many drug-related side effects, such as the pro-edema, pro-inflammatory, and pro-neurotoxic effects, as well as the potential risk to cause cerebral hemorrhage (29–31). Several studies have demonstrated that decreased PHE was observed after catheter aspiration and tPA thrombolysis either in animal models or in humans with ICH. Recently, the results from Minimally Invasive Surgery plus rt-PA for Intracerebral Hemorrhage Evacuation (MISTIE) Phase II demonstrated that hematoma evacuation could effectively reduce PHE, and administration of tPA for clot lysis following initial aspiration did not enhance edema formation compared to patients treated with clot aspiration only (18). Another prospective controlled trial from China enhanced the confidence in the MISTIE-II approach, showing better clinical outcomes with MISP therapy of acute ICH in combination with urokinase thrombolysis compared to open craniotomy (15, 16). Based on these above findings, we sought to investigate and compare the clinical values of uPA and tPA administrated following MISP in ICH patients.

The clinical significance of PHE remains poorly understood. It was reported the PHE volume could reach twofold to threefold the original volume of hematoma (32). So far, tPA is the most widely used fibrinolytic agent for IVH and ICH (33). However, increased evidence indicated that uPA was able to increase the rate of clot lysis without exacerbation of PHE, and improve outcomes in patients with spontaneous ICH (34, 35). Notably, Gaberel et al. demonstrated that, in contrast to tPA, uPA displayed a safer profile for intraventricular fibrinolysis following IVH regarding secondary inflammatory processes and neurotoxicity, as well as better neurological outcome in experimental models of IVH (36). In agreement with these above findings, our data suggested that uPA was associated with a slightly better effect on both short-term and long-term outcomes for fibrinolysis of ICH. After treatment with both agents, the hematoma ER in the uPA group was higher than that in the tPA group [82.6 ± 7.9% (uPA) vs 79.9 ± 9.5% (tPA), *p* = 0.332], showing a similar efficacy and a comparable fibrinolytic effect of tPA in ICH fibrinolysis. Further, we tested the volume of PHE on day 7 after operation, with 66.1 ± 22.4 mL in the uPA group, while 68.9 ± 19.1 mL in the tPA group. Although the volume was slight larger in the tPA group, the difference between both groups was not statistically significant.

The recurrence of ICH still represents the most severe complication of thrombolysis and a major obstacle to the generalization of thrombolytic therapy (37). For safety, in the clinical trial, the does and the first time of the administration of tPA were in accordance with MISTIE-II trial. 2/45 patients (4.4%) in the tPA group experienced a rebleeding event, in comparison with 2/41 patients (4.9%) in the tPA group, and the statistical analysis did not show the significant difference on the rebleeding incidence in the two groups (*p* = 0.924). The recurrence of ICH events observed in this study were similar to those reported in earlier clinical trials, notably the MISTIE phase 2 trial (38). Furthermore, no significant difference was observed in either the times of usage or catheter-retained time between both groups. In addition, the incidence of complications, including gastrointestinal bleeding, renal failure, intracranial infection, pulmonary infection, and epilepsy, was similar between both groups, and without a trend of exacerbation in those patients treated with surgery only reported in other clinical trials (39), suggesting a safety of these two fibrinolytics in ICH fibrinolysis. As for long-term outcomes, four indexes, including mortality, GOS, BI, and mRS, were used at 1 year postictus. The data in our study showed that all these four parameters between both groups were not statistically significant.

There are some limitations in the present study should be noted. First, the study was a retrospective study, and a larger prospective, randomized and controlled clinical trial is required to perform to confirm our findings. Second, a control group treated with hematoma aspiration only was absent from the design of the trial. As a result, we can not provide stronger evidence supporting the efficacy and safety of administration of uPA and tPA in ICH fibrinolysis. A further optimal design of study should be carried out. Finally, the inclusion and exclusion criteria in the clinical trial may not provide a complete and accurate evaluation of the clinical values of these two fibrinolytics in ICH fibrinolysis.

In conclusion, the clinical trial analyses and compares the differential effect of uPA and tPA administrated following MISP in patients with spontaneous basal ganglia ICH for the first time. Our findings offer the convinced evidence supporting that these two fibrinolytic agents are similar in the efficacy and safety for thrombolytic evacuation in combination with MISP in patients with spontaneous basal ganglia ICH. Based on these findings, further prospective, randomized, and double-blind clinical trials should be undertaken to confirm the full effects of these two fibrinolytics in ICH fibrinolysis.

## Author Contributions

The authors Participated in research design: YL, RY, ZL, BW, and LL. Conducted experiments: BT, XZ, JW, and LZ. Wrote or contributed to the writing of the manuscript: YL, RY, and LL.

## Conflict of Interest Statement

The authors declare that the research was conducted in the absence of any commercial or financial relationships that could be construed as a potential conflict of interest.
